# Comparing CenteringPregnancy® to standard prenatal care plus prenatal education

**DOI:** 10.1186/1471-2393-13-S1-S5

**Published:** 2013-01-31

**Authors:** Ingunn Benediktsson, Sheila W McDonald, Monica Vekved, Deborah A McNeil, Siobhan M Dolan, Suzanne C Tough

**Affiliations:** 1 Faculty of Medicine, University of Calgary, Canada; 2 Department of Paediatrics, University of Calgary, Calgary, Alberta, Canada; 3 Public Health Innovation and Decision Support, Alberta Health Services, Calgary, Alberta, Canada; 4 Department of Obstetrics & Gynecology and Women’s Health, Albert Einstein College of Medicine of Yeshiva University, Bronx, NY, USA; 5 Department of Community Health Sciences, University of Calgary, Calgary, Alberta, Canada

## Abstract

**Background**

There is significant evidence to support the importance of prenatal care in preventing adverse outcomes such as preterm birth and low infant birth weight. Previous studies have indicated that the benefits of prenatal care are not evenly distributed throughout the social strata. In addition, emerging evidence suggests that among particular populations, rates of preterm birth are unchanged or increasing. This suggests that an alternate care model is necessary, one that seeks to addresses some of the myriad of social factors that also contribute to adverse birth outcomes. In previous studies, the group prenatal care model CenteringPregnancy® had been shown to reduce adverse birth outcomes, but to date, no comparison had been made with a model that included prenatal education. This study sought to investigate whether any significant difference remained within the comparison groups when both models accounted for social factors.

**Methods**

This analysis was based on survey data collected from a prospective cohort of pregnant women through the All Our Babies Study in Calgary, Alberta.

**Results**

At baseline, there were significant differences between the comparison groups in their psychosocial health, with the women in the CenteringPregnancy® group scoring higher levels of depressive symptoms, stress and anxiety. At four months postpartum, the differences between the groups were no longer significant. Conclusions: These results suggest that CenteringPregnancy® can recruit and retain a demographically vulnerable group of women with a constellation of risk factors for poor pregnancy and birth outcomes, including poverty, language barriers and poor mental health. Post program, the rates of stress, anxiety and depression were similar to other women with more social and financial advantage. These findings suggest that CenteringPregnancy® may be a community based care strategy that contributes to improved mental health, knowledge, and behaviours to optimize outcomes for mothers and children.

## Background

The majority of prenatal care in Canada is provided by physicians or midwives in one-on-one visits, but approximately one-third of pregnant women in Canada choose to supplement this care by attending prenatal education classes [1]. In prenatal education classes, an instructor presents content that generally addresses child birth and maintaining a healthy pregnancy, but may also cover infant care and the early postpartum transition, to a group of pregnant women and their chosen support person [1,2]. CenteringPregnancy® group prenatal care, a new model that provides both medical care and education in a group setting, is emerging in Canada and other countries [3].

### Evidence regarding prenatal care

Prenatal care refers to the health services accessed by a woman during her pregnancy and involves careful monitoring of her health and her unborn child’s health throughout the pregnancy [4-6]. Depending on the care provider, the visits may also include information regarding safe practices and mental health [7]. Emerging evidence indicates that prenatal care may reduce adverse birth outcomes, however, the effects are not equally distributed across the social strata, and there is little evidence to show its effects on psychosocial outcomes [6,8,9]. Prenatal care is not sufficient to decrease the likelihood of adverse birth outcomes [6,10]. Rates of preterm birth and other adverse birth outcomes have remained relatively the same in spite of increased access to prenatal care programs [10,11]. This may indicate that a greater degree of intervention, or a different kind of intervention may be needed to improve overall birth outcomes. McLaughlin et al. [8] in their observations of low-income pregnant women found that comprehensive prenatal care resulted in an average increase in birth weight in the children of low-income mothers whereas standard prenatal care had little effect. Psychosocial outcomes related to the prenatal period are difficult to assess and to understand their full impact, although they can influence maternal and child health across the life course [12,13].

### Evidence regarding prenatal education

A recent systematic review reported that the effects of prenatal education classes for women and children was difficult to determine due to the limitations of research to date (e.g. small sample sizes, varying quality, lack of randomized controlled trials)[14]. Randomized controlled trials of prenatal education are few and far between, and such trials are difficult to conduct given how widespread prenatal education is and the challenges with randomization (e.g. lack of participant willingness and adherence to randomization) [14]. While randomized controlled trials are emerging in the area of prenatal education, they tend to focus on targeted initiatives such as improvements in breastfeeding initiation [15]; knowledge, nutrition, and health behaviours [16]; psychosocial well-being [17,18]; and co-parenting, parenting and infant regulation [17]. With regard to general prenatal education initiatives, one observational study found no association between attendance at prenatal education classes and infant birth weight, maternal weight gain, or reduction in smoking [19]. Other observational studies have found associations between attendance at childbirth education and continued breastfeeding at 6 months [20,21], breastfeeding initiation [22], and introduction of solid foods within recommended guidelines [23].

It is difficult to determine whether such associations are a result of prenatal education or due to the characteristics of women who attend prenatal education classes versus those who do not attend. Women who attend prenatal education are more likely to be first-time mothers [1]. Canadian women under 20 years of age are more likely to attend prenatal classes than all other age groups [1,24], but older women are more likely to attend prenatal education classes than younger women when it is their first live birth [19,25]. Other North American research indicates that the characteristics of women more likely to attend prenatal education classes are Caucasian [26], some post-secondary education [26], married [26], not living in a low-income household [1,26,27], and less likely to smoke before pregnancy [19].

### Evidence regarding CenteringPregnancy®

Due to the emerging nature of CenteringPregnancy®, randomized controlled trials have been easier to conduct on CenteringPregnancy® than on prenatal education classes. Research evidence from two such trials indicated better outcomes, including improved prenatal knowledge, greater satisfaction with care, a higher likelihood of having an adequate number of prenatal visits, decreased risk of preterm birth, and a greater readiness for delivery and baby care, for women in CenteringPregnancy® compared to those in individual prenatal care [28,29]. Studies comparing CenteringPregnancy® to individual care using a less rigorous cohort design [30-35] are inconclusive, with inconsistency in the findings of these studies. Results from a recent systematic review and meta-analysis comparing group and individual prenatal care found that women who attended group prenatal care were less likely to give birth prematurely and more likely to breastfeed. As noted by the authors, however, the results lack generalizability due to the inconsistency in the literature examined [36].

### Comparisons of CenteringPregnancy® and prenatal education

While there are some similarities between CenteringPregnancy® and prenatal education classes (e.g. similar goals, the group setting, and some content), there are also some important differences [2]. Women in CenteringPregnancy® receive prenatal care visits and education concurrently and at the same location, sessions begin earlier within the first or second trimester, and discussions are directed by those in the group [2]. Prenatal education classes, on the other hand, are held separate from prenatal care visits, classes generally begin later (i.e. in the third trimester), and discussions are led by an instructor [2].

To date, the literature that compares quantifiable aspects of Centering Pregnancy to alternative care models focuses only on the comparison of CenteringPregnancy® to individual prenatal care with a health professional. No studies have compared CenteringPregnancy® to individual prenatal care plus prenatal education, either in terms of the characteristics of women attending or their outcomes. The objective of this analysis was to compare women in CenteringPregnancy® to women in individual prenatal care plus prenatal education to determine if they differed according to demographic characteristics, psychosocial well-being (social support, depressive symptoms, anxiety, and stress), type of information received during prenatal care visits, health behaviours during pregnancy, and use of community resources. Those planning prenatal programs will be able to use such information to understand the profile of those attending different programs or models of care as well as the potential impact of such programs.

## Methods

This analysis was based on survey data collected from a prospective cohort of pregnant women through the All Our Babies Study in Calgary, Alberta, Canada.

### Models of prenatal care and education

The All Our Babies Study was conducted in Calgary, Canada (2008-2011), and is described in detail elsewhere [37,38]. This analysis examined participants who reported participating in local prenatal education classes as compared to those who participated in a CenteringPregnancy® program. Calgary prenatal education classes typically include a maximum of about 12 pregnant women (plus their support person) and 12 to 20 hours of education are provided. Women must pay a fee to attend most prenatal education classes in Calgary. Prenatal education classes are provided through the provincial health region at a variety of health care locations throughout the city as well as through private providers.

The CenteringPregnancy® program was co-facilitated by family physicians from a low-risk maternity clinic and prenatal educators from the provincial health region. Groups of 8 to 12 pregnant women at a similar stage of pregnancy met for ten CenteringPregnancy® sessions that lasted two hours each [39,40]. During the first part of each session, women received an individual physical assessment from the physician in the group space and also performed self-care activities, such as measuring and recording their own blood pressure and weight [40]. This was followed by a discussion that focused on general topics related to pregnancy, childbirth, or parenting [40]. The discussions were facilitated by a family physician and educator, encouraging those within the group to provide input on the specific content [40]. Women also had opportunities to interact socially with each other during the sessions [40]. Some groups allowed support people at all of the sessions while other groups only allowed support people for certain sessions. The CenteringPregnancy® program was offered in a region of the city that contained a higher proportion of immigrants and those with lower socioeconomic status [41]. In Calgary, ‘Birth and Babies’ prenatal classes are offered for a fee, however, a fee waiver is available for low income women. For this study, CenteringPregnancy® was provided at no cost to pregnant women and was offered within the context of usual medical care. Prenatal medical care in Canada is funded through a public health care system. At the time of the study, site accreditation for the CenteringPregnancy® program had not yet been obtained, however all providers were trained through the Centering Healthcare Institute (https://www.centeringhealthcare.org).

### Recruitment and sample

Women in the All Our Babies Study were recruited using multiple strategies including local health care offices, community posters, the Alberta Health Services website, and Calgary Laboratory Services. Women were also recruited from the CenteringPregnancy® program offered through the Maternity Care Clinic. Women were eligible to participate if they were less than 25 weeks gestation at the time of recruitment, receiving prenatal care in Calgary, and able to complete the questionnaires in English. Approximately 3,300 women participated in the All Our Babies Study. Two groups of women were compared in the present study: (1) women who participated in standard individual prenatal care and the Birth and Babies prenatal education class (n=619) and (2) women who participated in CenteringPregnancy® (n=106). Although we did not exclude women from either group who accessed prenatal education other than that offered in each respective group, we did exclude one woman in CenteringPregnancy® who also reported participating in the Birth and Babies prenatal education class from the analysis.

### Data collection and measurement

Data were collected through three mailed surveys, in early pregnancy (<25 weeks gestation; baseline), between 34 and 36 weeks gestation, and 4 months postpartum. Of those who were eligible, 85% completed at least one questionnaire. The response rate across the three time points was 74%, with pregnancy loss or lost to follow-up constituting the most common reasons for attrition. The three surveys included questions on demographic characteristics, pregnancy history, service utilization, nutrition and exercise practices, health, psychosocial factors (e.g. social support, depressive symptoms, anxiety, stress), lifestyle and life history, and breastfeeding.

Standardized scales were used to measure psychosocial factors (Table 1). Recall of information received during prenatal care visits was measured during late pregnancy by asking women to indicate if they received advice on eight topics (nutrition, alcohol consumption, weight gain, prescription/non-prescription drugs, vitamin/mineral supplements, exercise, working, and smoking). Health behaviours were assessed by measuring maternal nutrition, consumption of alcohol and cigarettes, and maternal intentions and practices around infant feeding and health. At 4 months postpartum, breastfeeding, feeding, and infant health data were collected, as well as usage of local community resources.

**Table 1:** Standardized tools for psychosocial factors assessed in the AOB study

Psychosocial Factors	Standardized Tool	Scoring	Baseline (<25wks)	34-36 wks	4 months
Depressive Symptoms	Edinburgh Postnatal Depression Scale (EPDS) [58]	10 item questionnaire. Each item rated on a 4-point Likert scale from 0-3. After reverse scoring for some items, a total score is derived (range 0-30). Higher scores reflect increased depressive symptoms. Standard cut-off for risk of major depression as per the literature: 13 and above.	x	x	x

Anxiety	State-Trait Anxiety Inventory (state anxiety scale; SAI) [59]	20 item questionnaire. Each item rated on a 4-point Likert scale from 1-4. After reverse scoring for some items, a total score is derived (range 20-80). Higher scores reflect increased anxiety symptoms. We used an established cut-off of 40 or more to classify women as anxious.	x	x	x

Stress	Perceived Stress Scale (PSS) [60]	10 item questionnaire. Each item rated on a 5-point Likert scale from 0-4. After reverse scoring for some items, a total score is derived (range 0-40). Higher scores reflect increased stress symptoms. A cut-off at the 80^th^ percentile of the sample distribution was used to classify women as stressed.	x	x	x

Social Support	Medical Outcomes Study (MOS) Social Support Scale [61]	19 item questionnaire. Each item rated on a 5-point Likert scale from 1-5. Scoring algorithms derive subscale scores and a total score (range 0-100). Higher scores reflect increased perception of social support. We used an established cut-off of 69 or less to classify women as having low perceived social support.	x	x	x

### Statistical analysis

Frequencies and percentages were calculated for categorical variables and means and standard deviations (or medians and interquartile range; IQR) for any continuous variables. Differences between the groups were analyzed using chi-square tests for categorical variables and Fischer’s exact test when expected cell frequencies were less than 5. All psychosocial scores from standardized scales were dichotomized to classify women as either reporting excessive symptomatology (vs. not) on the respective scale (e.g., high stress, low social support). Finally, classification of change from high to low symptomatology (vs. no change/low to high) was operationalized as a categorical variable derived from baseline and 4 months postpartum.

## Results

The median number of sessions attended by women in CenteringPregnancy® was 6 (IQR=8). Women in CenteringPregnancy® and standard individual care plus prenatal education were similar with regard to marital status and maternal age, but differed on a number of other demographic characteristics (Table 2). Women in the CenteringPregnancy® group were more likely to have completed a lower level of education, have a lower household income, be of non-Caucasian ethnicity, be born outside of Canada, and to primarily speak a language other than English in the home. Women in the CenteringPregnancy® group were also more likely to have had their first prenatal appointment at a walk-in clinic rather than with a family physician in an appointment based office, a physician at a low risk maternity clinic, an obstetrician, or a midwife.

**Table 2:** Demographic characteristics of participants: group comparisons

Demographic characteristic	CP^a^ n=106 n (%)	PE^a^ n=619 n (%)	*p*-value
Single	5 (4.9)	36 (5.8)	0.696
Maternal age at delivery			0.101
<25	10 (9.6)	30 (5.2)	
25-34	81 (77.9)	441 (76.6)	
≥35	13 (12.5)	105 (18.2)	
High school education or less	17 (16.2)	33 (5.30)	<0.001
Annual household income < $40,000	14 (13.6)	32 (5.3)	0.002
Non-Caucasian ethnicity	41 (39.4)	132 (21.3)	<0.001
Not born in Canada	31 (29.5)	120(19.4)	0.018
Speak a language other than English in the home	26 (25.0)	54 (8.7)	<0.001
First prenatal appointment at a walk-in clinic	26 (25.5)	59 (10.2)	<0.001

^a^ CP= CenteringPregnancy®; PE=Prenatal Education (plus individual prenatal care)

There were significant differences in psychosocial health variables between the CenteringPregnancy® group and the prenatal education group at baseline, with a greater proportion of women in the CenteringPregnancy® group having lower levels of social support and higher levels of depressive symptoms, stress, and anxiety (Table 3). At 4 months postpartum, the differences between the two groups were no longer significant. Analysis of the change in psychosocial health variables indicated that women in CenteringPregnancy® were significantly more likely than women in the prenatal education group to report improvements in symptoms for depression, stress, and anxiety, but not for social support.

**Table 3:** Psychosocial health: group comparisons

Variable	CP^a^ n=106 n (%)	PE^a^ n=619 n (%)	*p*-value
Lower social support (<70 on MOS Social Support Scale)			
Baseline (<25 wks)	20 (19.4)	60 (9.7)	0.004
4 months postpartum	16 (17.6)	75 (12.2)	0.154
Improved perception of social support	7 (7.8)	28 (4.6)	0.195
Depressive symptoms (10 or higher on EPDS)			
Baseline (<25 wks)	26 (25.0)	90 (14.6)	0.008
4 months postpartum	16 (17.4)	28 (4.6)	0.250
Improved depressive symptoms^c^	15 (16.3)	52 (8.5)	0.017
Higher stress (≥18 on PSS)			
Baseline (<25 wks)	33 (31.4)	138 (22.5)	0.049
4 months postpartum	12 (13.2)	96 (15.7)	0.541
Improved stress^d^	22 (24.2)	84 (13.9)	0.017
Higher anxiety (≥40 on SAI)			
Baseline (<25 wks)	30 (30.3)	91 (15.4)	<0.001
4 months postpartum	12 (13.0)	87 (14.2)	0.759
Improved anxiety^e^	19(22.1)	51 (8.7)	<0.001

^a^ CP= CenteringPregnancy®; PE=Prenatal Education (plus individual prenatal care)

^b^ Improvement operationalized as moving from category reflecting low social support to high social support

^c^ Improvement operationalized as moving from category reflecting high depression to low depression

^d^ Improvement operationalized as moving from category reflecting high stress to low stress

^e^ Improvement operationalized as moving from category reflecting high anxiety to low anxiety

In terms of women’s recall of information received at their prenatal visits during their pregnancy, as assessed in the third trimester, similar proportions in the CenteringPregnancy® group and the prenatal education group recalled receiving information on most topics. Women in the CenteringPregnancy® group were more likely to recall receiving information during pregnancy on nutrition, alcohol consumption, and smoking or second hand smoke than those in the prenatal education group (Table 4). Comparison of health behaviours between the two groups are seen in Table 5. There were no differences between women in the CenteringPregnancy® group and those in the prenatal education group with respect to meeting the daily recommended intake of food group servings. Women in CenteringPregnancy® were less likely to consume alcohol before their pregnancy yet there were no differences in pre-pregnancy smoking rates between the two groups. In comparing substance use rates during and after pregnancy among pre-pregnancy users, results showed that women in CenteringPregnancy® were less likely to have stopped smoking during pregnancy and after birth. There were no group differences in alcohol consumption rates during pregnancy or in the postpartum among pre-pregnancy drinkers. In terms of infant feeding, there were no differences between the two groups in plans to breastfeed and breastfeeding initiation. However, women in the CenteringPregnancy® group were less likely to still be breastfeeding at 4 months postpartum and were more likely to have started their infants on solid food at or prior to 4 months of age than those in the prenatal education group. There were no differences between the two groups in physician check-ups and vaccinations for their infants.

**Table 4:** Information received during prenatal visits: group comparisons

Recalled receiving information on:	CP^a^ n=106 n (%)	PE^a^ n=619 n (%)	*p*-value
Appropriate amount of weight gain	89 (84.0)	473 (76.4)	0.085
Exercise or active living during pregnancy	83 (78.3)	434 (70.1)	0.085
Nutrition	92 (86.8)	453 (73.2)	0.003
Taking vitamins or mineral supplements	92 (86.8)	543 (87.7)	0.789
Taking prescription or non-prescription drugs	75 (70.8)	430 (69.5)	0.790
Alcohol consumption during pregnancy	77 (72.6)	350 (56.5)	0.002
Cigarette smoking and second hand smoke	71 (67.0)	299 (48.3)	<0.001
Working during pregnancy	65 (61.3)	343 (55.4)	0.257

^a^ CP= CenteringPregnancy®; PE=Prenatal Education (plus individual prenatal care)

**Table 5:** Health behaviours: group comparisons

Health behaviour	CP^a^ n=106 n (%)	PE^a^ n=619 n (%)	*p*-value
Meeting recommended intake of:			
Meat and alternatives (at least 2 servings)	81 (76.4)	424 (68.6)	0.106
Milk and alternatives (at least 2 servings)	88 (82.2)	537 (86.9)	0.198
Fruits and vegetables (at least 7 servings)	9 (8.6)	62 (10.0)	0.645
Grain products (at least 6 servings)	28 (26.4)	169 (27.3)	0.850
Alcohol consumption			
Before pregnancy	77 (74.0)	544(87.9)	<0.001
While pregnant^b^	52 (68.4)	318 (58.5)	0.097
Since birth^b^	50 (75.8)	390 (75.1)	0.913
Smoking			
Before pregnancy	26 (25.0)	117 (18.9)	0.151
While pregnant^c^	19 (73.1)	53 (45.3)	0.01
Since birth^c^	11 (57.9)	22 (18.8)	0.001
Infant feeding			
Planned to breastfeed	102 (96.2)	606 (97.9)	0.294
Initiated breastfeeding	95 (100.0)	612 (99.0)	1.000
Still breastfeeding at 4 months postpartum	70 (73.7)	520 (85.0)	0.006
Started solid foods by 4 months postpartum	23 (22.1)	36 (5.9)	<0.001
Infant health			
Baby has been for a doctor check-up since birth	104 (99.0)	605 (97.9)	0.705
Baby has received vaccinations	100 (95.2)	570 (92.2)	0.275

^a^ CP= CenteringPregnancy®; PE=Prenatal Education (plus individual prenatal care)

^b^ among pre-pregnancy drinkers

^c^ among pre-pregnancy smokers

Finally, women in the prenatal education group were more likely to access a number of community resources since the birth of their baby, including local community health centres, a local parenting resource book, informal mom and tot groups, local libraries, and parenting classes than those in the CenteringPregnancy® group (Table 6). Women in the prenatal education group were also more likely to access a wider range of community resources.

**Table 6:** Use of community resources at 4 months postpartum: group comparisons

Community resource(s) used	CP^a^ n=106 n (%)	PE^a^ n=619 n (%)	*p*-value
Local community health centre	44 (41.5)	338 (54.6)	0.013
“Growing Miracles” parenting resource book	38 (35.8)	286 (46.2)	0.048
Informal mom and tot groups	21 (19.8)	263 (42.5)	<0.001
Local fitness, recreation or leisure centre	36 (34.0)	256 (41.4)	0.151
Local library	24 (22.6)	245 (39.6)	0.001
Television show about parenting	24 (22.6)	195 (31.5)	0.066
Free parenting magazine/newspaper	22 (20.8)	182 (29.4)	0.067
Parenting classes	20 (18.9)	205 (33.1)	0.003
Parenting group on the internet	14 (13.2)	118 (19.1)	0.149
Local church or spiritual leader, mentor, or organization	18 (17.0)	108 (17.4)	0.907
Wider variety of community resources (5 or more resources)	18 (17.0)	234 (37.8)	<0.001

^a^ CP= CenteringPregnancy®; PE=Prenatal Education (plus individual prenatal care)

## Discussion

The findings of this analysis indicate that there are differences, both prenatally and in the postpartum period, between the women who received individual physician care and chose to attend prenatal education classes and those who chose to take part in CenteringPregnancy®. Women who attended CenteringPregnancy® had a different demographic profile than women in the standard care and prenatal education group in that they had completed less education, were of lower income, and were more likely to be non-Caucasian and foreign-born, and to speak a language other than English in their home. These differences could reflect the recruitment site for women in CenteringPregnancy®, which was located in a neighborhood with high representation of immigrant groups and lower socioeconomic status (SES) families [41], They were also more likely to use a walk-in clinic for their first prenatal visit, suggesting potential difficulties in becoming engaged in the health care system. Previous research has indicated that women of lower SES are less likely to have a regular family physician and are more likely to seek routine care from an emergency or walk-in facility [8,26].

At baseline, women participating in CenteringPregnancy® were also more likely to have poorer psychosocial health than women attending prenatal education. However, at 4 months postpartum, there were no longer any significant differences in any of the psychosocial health variables between the two groups. An analysis of change in psychosocial health status between the two time points indicated CenteringPregnancy® women were more likely to have improved psychosocial health across time. Although the change analysis for improvement in social support was not significant, there was a greater proportion of women in CenteringPregnancy® than in the comparison group that reported an improvement in their perception of social support. The non-significant result could be due to low power or insensitivity in the social support measure used (i.e., total social support) and its cut-off. Indeed, established cut-offs as per the literature are developed among the general population and not for pregnant women *per se.* Improved psychosocial health is particularly remarkable given the financial, language and social disadvantage this group of women reflected. However, given that the Centering Pregnancy program places significant emphasis on building social support within the group, this data suggest that participation in CenteringPregnancy® helped to improve women’s psychosocial health, which aligns with findings reported in a recent study in the area [42]. These findings could have important implications given that maternal mental health during the prenatal period is an important contributor not only to an infant’s birth status, but has also been correlated with the continued health and development of the infant throughout their childhood [30,43-49]. Current guidelines from the Society of Obstetrics and Gynaecology of Canada also emphasize the importance of caring for psychosocial health during pregnancy [7], and these findings indicate that CenteringPregnancy® may provide care that improves women’s psychosocial well-being.

An important finding gleaned from the comparisons pertains to information received during pregnancy. When asked in late pregnancy, women in CenteringPregnancy® reported that they were more likely to have received information on nutrition, alcohol, and smoking than women in prenatal education, suggesting that women may receive more information on these topics in CenteringPregnancy® or may more readily retain the information gained in CenteringPregnancy®. Although difficult to tease out, further analyses controlling for markers of maternal literacy would be of value. Further exploration of these findings needs to also consider issues of timing, uptake, and delivery of information as these factors may influence information recall differences. Indeed, CenteringPregnancy® is more flexible than regular classes, and this type of structure may be better suited to retention and uptake of information.

In terms of health behaviours, a greater proportion of women in CenteringPregnancy® continued to smoke during their pregnancy; given the psychosocial health and SES profile of the CenteringPregnancy® women this finding may be due to confounding factor rather than insufficient support for smoking cessation. Rates of alcohol consumption during and after pregnancy among pre-pregnancy drinkers were similar in the two groups, which also may reflect the demographic profile and differences in attitudes towards alcohol between the two groups, as the northeast quadrant of Calgary has a higher population of immigrants and people belonging to cultural groups who abstain from alcohol [41]. Indeed, the demographic profile of the CenteringPregnancy® women provides context for interpreting the results for a number of the comparisons. Women of lower SES have been found to be less likely to initiate breastfeeding and less likely to have positive nutritional habits [26,27,49,51,52]. However, despite differences in their demographic profiles, women in CenteringPregnancy® and women in prenatal education had similar nutritional habits as well as similar levels of intentions to and initiation of breastfeeding. If the women of the CenteringPregnancy® group are assumed to have a similar baseline to other groups of vulnerable women that are reported in the literature, then it is possible that the lack of difference in breastfeeding initiation and nutritional habits could be attributed at least in some part to the prenatal care program. Nevertheless, women in CenteringPregnancy® were less likely to still be breastfeeding at 4 months postpartum and were more likely to start solid foods earlier than is considered optimal [23,53], consistent with earlier studies that established, that women of lower SES are less likely to breastfeed and engage in optimal feeding practices [26,27,49,51,52]. Cultural practices may also be a factor to consider. For these reasons, without a demographically matched group or analytic control, it is impossible to separate the effects of CenteringPregnancy® or prenatal education from that of socioeconomic, cultural, and sociodemographic factors.

Women in prenatal education were more likely to use a number of community resources in the postpartum period and overall more likely to access a wider range of community resources than were women in CenteringPregnancy®. This finding is challenging to interpret, as it could indicate that women in CenteringPregnancy® perceived less of a need to access community resources as they had developed social and informational networks through CenteringPregnancy®, or that as a consequence of accumulated poorer mental health, low proximity to services, transportation barriers, and financial and work related circumstances, these women were less likely or able to access these resources.

### Limitations

Considering the differences in the sociodemographic profile of the two groups, we are unable to determine if any differences in late pregnancy or postpartum variables are due to the model of care and education or socio-demographics and baseline differences between women in CenteringPregnancy® and those in the comparison group. Further analyses using multivariable regression models to adjust for socioeconomic and sociodemographic confounding variables are clearly warranted. In addition, many of the outcomes point to areas that need further exploration, which may not be possible using the survey data available. In particular, the positive change seen in the psychosocial domain warrant further exploration. Not all issues related to maternal mental health and well-being were explored in this study. In particular, the idea of empowerment was not addressed in this study, due to a lack of available data. If a perceived sense of empowerment can be understood to have a positive effect on other psychosocial health variables, such as stress or anxiety, then future research should attempt to identify disentangle its effects. Empowerment should be explored qualitatively, so that researchers can identify what lived experiences of prenatal care help a woman gain a sense of empowerment, and how such a sense enhances her capacity to cope in both the prenatal and postpartum periods.

## Conclusion

The prenatal time period is an important time influencing child development [12,54-56], and programs that aim to improve maternal and child health outcomes need to be developed and studied in relation to existing programs. Although emerging evidence suggests that CenteringPregnancy® improves outcomes when compared to standard prenatal care [28,29,42,57], its effectiveness in comparison to a more comprehensive form of prenatal care and education has not yet been fully explored. The results of this study suggest that CenteringPregnancy® can recruit and retain a demographically vulnerable group of women with a constellation of risk factors for poor pregnancy and birth outcomes, including poverty, language barriers and poor mental health. Post program, the rates of stress, anxiety and depression are similar to other women with more social and financial advantage. These results generate hypotheses to be further tested in additional analyses and future studies. Although further research is warranted, these findings suggest that CenteringPregnancy® may be a community based care strategy that contributes to improved mental health, knowledge, and behaviours to optimize outcomes for mothers and children.

## Competing interests

The authors declare that they have no competing interests.

## Authors’ contributions

SCT is responsible for the overall integrity, progress and timely completion of the AOB study. DAM and SMD participated in the design of the study. SWM helped analyze the data. MV contributed to drafting the manuscript. All authors contributed to interpretation of the results. IB performed the literature review and drafted the manuscript. All authors have read and approved the final manuscript.

## Declarations

This article has been published as part of *BMC Pregnancy and Childbirth* Volume 13 Supplement 1, 2013: Preterm Birth: Interdisciplinary Research from the Preterm Birth and Healthy Outcomes Team (PreHOT). The full contents of the supplement are available online at http://www.biomedcentral.com/bmcpregnancychildbirth/supplements/13/S1.

All of the publication fees will be funded by the Preterm Birth and Healthy Outcomes Team Interdisciplinary Team Grant (#200700595) from Alberta Innovates - Health Solutions, formerly the Alberta Heritage Foundation for Medical Research.
